# Involving lived experience in regional efforts to address gambling-related harms: going beyond ‘window dressing’ and ‘tick box exercises’

**DOI:** 10.1186/s12889-024-17939-7

**Published:** 2024-02-05

**Authors:** Catherine L. Jenkins, Thomas Mills, James Grimes, Colin Bland, Paula Reavey, Jane Wills, Susie Sykes

**Affiliations:** 1 Institute of Health and Social Care, London South Bank University, London, UK; 2 BetKnowMore, London, UK; 3 School of Applied Sciences, London South Bank University, London, UK

**Keywords:** Lived experience, Public engagement, Public involvement, Gambling-related harms, Harms reduction

## Abstract

**Background:**

Lived Experience (LE) involvement has been shown to improve interventions across diverse sectors. Yet LE contributions to public health approaches to address gambling-related harms remain underexplored, despite notable detrimental health and social outcomes linked to gambling. This paper analyses the potential of LE involvement in public health strategy to address gambling-related harms. It focuses on the example of a UK city-region gambling harms reduction intervention that presented multiple opportunities for LE input.

**Methods:**

Three focus groups and 33 semi-structured interviews were conducted to hear from people with and without LE who were involved in the gambling harms reduction intervention, or who had previous experience of LE-informed efforts for addressing gambling-related harms. People without LE provided reflections on the value and contributions of others’ LE to their work. Data analysis combined the Framework Method with themes developed inductively (from people’s accounts) and deductively (from the literature, including grey literature).

**Results:**

Four themes were identified: (1) personal journeys to LE involvement; (2) the value added by LE to interventions for addressing gambling-related harms; (3) emotional impacts on people with LE; and (4) collective LE and diverse lived experiences. Two figures outlining LE involvement specific to gambling harms reduction in the UK, where public health efforts aimed at addressing gambling-related harms coexist with industry-funded programmes, are proposed.

**Conclusions:**

Integrating a range of LE perspectives in a public health approach to gambling harms reduction requires local access to involvement for people with LE via diverse routes that are free from stigma and present people with LE with options in how they can engage and be heard in decision-making, and how they operate in relation to industry influence. Involving LE in gambling harms reduction requires enabling people to develop the affective and critical skills necessary to navigate complex emotional journeys and a challenging commercial and policy environment.

## Plain English summary

Lived Experience (LE) refers to what people know from their experience. It goes beyond formal education or professional experience to provide unique insights that can improve public health research and service provision. This paper discusses the role of LE in a UK city-region government’s gambling harms reduction intervention.

Internal and external stakeholders, including people with and without LE, reflected on the value of LE and challenges to its effective involvement in interviews and focus groups. The evaluation team summarised the data collected into four themes: (1) personal journeys to LE involvement (people have unique journeys to involvement in the gambling harms reduction sector); (2) the value added by LE to interventions for addressing gambling-related harms (specifically the activities of supporting people and driving social change); (3) emotional impacts on people with LE (both positive and negative); and (4) collective LE and diverse lived experiences (i.e., the importance of balancing a cohesive LE community with representing the diversity of people and their experiences). The paper concludes that LE should be understood as many diverse lived experiences. Resources to support people to manage the emotional tensions raised by LE activities are needed, alongside routes to LE involvement that facilitate self-development and improve representation in positions of authority and decision-making.

## Background

### Lived experience in public health

Lived Experience (LE), or “knowing from experience”, refers to people’s direct or indirect experiential knowledge of an issue or service [1]. LE involvement in mental health services and research is longstanding [2, 3] and recognised as important in public health [4–7], where it echoes established approaches to public involvement and asset-based community development [8]. The emphasis on knowledge in LE distinguishes it from Patient and Public Involvement and Engagement (PPIE), although PPIE can be an engagement route for involving ‘experts by Lived Experience’, or ‘people with Lived Experience’ (PLE). The developing research base on LE involvement in public health intervention design and delivery highlights its importance for ensuring relevance and practical impact [9, 10], and for reducing the risk that an intervention may unintentionally exacerbate harms [1, 9].

Gambling-related harms (GRH) are complex and span a continuum [11]. They include harms to relationships, resources, and health [12], such as intimate partner violence [13], crime [14], costs to the economy [15], and suicide [16], with wide-ranging consequences for individuals, families, communities and society [17]. Research is increasingly revealing the extent of commercially-driven normalisation of gambling and its harmful products, as well as the tactics of the gambling industry, which echo other harmful industries, in circumventing regulation and shaping associated discourses [18]. LE involvement in GRH research and practice is growing, with further research required to more fully understand its contribution [12, 19].

This paper explores the value of LE as part of a regional public health intervention for addressing GRH that incorporated multiple opportunities for input by PLE. It provides insights into different facets of LE involvement in the gambling harms reduction sector and examines the contributions of LE, as well as the tensions that can arise in practice.

### Lived experience in the gambling sector

Gambling has been discussed as a public health issue for more than twenty years [12, 20, 21], yet there is no national public health framework for GRH reduction in the UK, and a comprehensive, socio-ecological public health perspective on gambling remains nascent [22–24]. The development of a robust public health approach is further complicated by the complexity of the UK policy environment, in which the influence of the gambling industry, rather than statutory public services, is well-entrenched in guiding health promotion, prevention and treatment activities [24]. In the absence of UK government leadership, GRH continues to be a ‘niche’ issue, with local initiatives sometimes implemented without the resources to ensure sustainability [25].

Engaging PLE in facilitating recognition and awareness of GRH is an emerging area of study [12, 19, 26, 27] and in the meantime, GRH treatment and education are funded and, in some cases, directly provided by the gambling industry, rather than statutory public health bodies [18, 28]. The development of LE involvement in the gambling sector is distinct because of this policy context, which is also important for understanding the barriers that PLE face when they get involved in the sector.

LE has much to offer public health policy analysis and development [29], but LE-led advocacy work on gambling reform contends with outdated UK legislation around a public health response to GRH. LE-led grassroots campaigns in the UK and further afield [24, 33] have sought to challenge the influence of the gambling industry over education and harms messaging framed as “responsible” or “safer” gambling [30] that push the responsibility for GRH onto individuals (“problem gamblers”) [31, 32]. The delayed UK Government review of the Gambling Act 2005 – to render gambling regulation fit-for-purpose in today’s digital world [33] – has so far been limited to a white paper [34] and various consultations, but this does recognise a need for public health-framed messaging and an end to industry-funded campaigns. Regulatory developments and product innovation and diversification in the industry [21] mean it is timely to advance applications of LE within a public health approach specific to gambling [11, 35], and in the context of public health discourse around commercial determinants of health [36].

Research suggests that strategies to address GRH are more effective when they incorporating LE-based perspectives [12]. In particular, PLE can contribute unique knowledge based on their experiences of how the gambling industry, its products, practices, and discourse, harms people [37]. LE involvement has already sought to influence language and inform service development, as in the significant pushback from PLE in response to the dominant framings of the industry based on the way in which such narratives shift the focus away from the problematic nature of addictive gambling products and onto individuals [37]. However, the lack of evidence-based frameworks for appropriately engaging PLE in GRH reduction activities, which can be attributed to the sensitivity and stigma surrounding gambling, has only recently begun to be addressed [38]. As online gambling continues to be a target area for growth and precision marketing, it is imperative that public health researchers and practitioners engage with communities to raise awareness of GRH [1] and develop their own and others’ critical health literacy [39] specific to gambling.

### Communities addressing gambling harms: a public health intervention for gambling-related harms reduction

The Communities Addressing Gambling Harms (CAGH) intervention was delivered across a city-region in the UK through 12 diverse community projects and a LE advisory panel (LEAP) that advised the community projects. It aimed to facilitate regional understanding of and action on GRH, and presented several opportunities for LE involvement: from advising the city-region government and the community projects on GRH reduction interventions, through to more active involvement in the projects (e.g., as educators or peer support workers).

A process evaluation of the CAGH intervention was approved by university ethics no. [anonymised] and the main evaluation is described in detail elsewhere [40]. The current paper broadens its focus beyond the CAGH intervention to investigate LE involvement in the GRH reduction sector more generally, in recognition that LE in CAGH is influenced by experiences external to it. This was necessary to fully understand LE in CAGH and reflected the complex reality of LE involvement on-the-ground, with PLE in CAGH often engaging in gambling harms reduction activities outside of the intervention.

A PPIE panel of public representatives contributed to the evaluation at every stage, including providing insight into local developments; advising on the data collection tools; assisting with recruitment; and theorising emergent data. The PPIE panel was recruited via a public call for expressions of interest. Two PPIE panel members are co-authors of this paper.

## Methods

### Sample

Three groups of people who were involved in the intervention were sampled to inform the evaluation:

1. People with LE (LEAP members, PPIE panel members, project staff, advisors): n-14.
2. Project staff without (declared) LE: n-14.
3. Senior stakeholders without (declared) LE: n-4.

Sampling was based on individuals’ potential ‘information power’, or diversity of experience [41, 42]. The ‘without LE’ status of project staff and senior stakeholders is qualified by ‘declared’ above, in recognition that while these groups contained no people who self-reported LE specific to GRH, distinctions between PLE and people without LE are not clear-cut. Further details of the sample are reported below:

1. **People with LE (PLE)**: People with LE of their own or someone else’s gambling, i.e., as an Affected Other (AO). Ensuring the terms used to describe the expertise and experiences of PLE reflect their preferences is important [10]. PLE does not define people by their LE, but recognises LE as an ongoing part of people’s expertise and daily life [26]. Here, LE relevant to GRH is defined as ‘Having personally experienced the suffering and destructive consequences of gambling related to own or someone else’s gambling’ [26]. This definition explicitly includes AOs – people in an individual’s social network who are affected by the individual’s LE or LE testimony [11, 35, 43].

PLE included people internal to the community projects delivering the intervention locally, and people external to the intervention but who had relevant expertise. Recruitment to the LEAP was via advocacy organisations and word-of-mouth. Recruitment into the research was via the study PPIE panel and snowballing: participants self-identified as PLE and were required to have been in self-reported recovery for at least 12 months prior to the time of the intervention. Ages of participants ranged from 33 to 69 years old; eight were male and six were female; and except for one Indo-Caribbean participant, all were White British.

2. **Project staff without LE (PS)**: People internal to the community projects in Voluntary, Community, Faith and Social Enterprise (VCFSE) organisations that were leading on or administering the intervention locally. Recruitment of PS was via the evaluation team contacting PS they had met at online and in-person meetings held as part of the CAGH intervention.
3. **Senior stakeholders (SS)**: Public health professionals (people who commission or implement gambling harms reduction programmes), representing posts at local, regional and national levels. The evaluation team contacted SS independently, after email addresses were provided by CAGH intervention leads.

Compared to PS and SS, PLE held complex and varied roles in the intervention and contributed to the evaluation in multiple ways. The involvement of PLE is highlighted and summarised in Table 1.

**Table 1:** Summary of PLE roles in the intervention and contributions to the evaluation

Identifier	Intervention role	Evaluation contribution
PLE1	LEAP	2x interviews; 1x focus group; PPIE panel
PLE2	LEAP	1x interview
PLE3	LEAP	1x interview, 3x focus groups
PLE4	LEAP	1x interview; PPIE panel
PLE5	LEAP	1x interview; 1x focus group
PLE6	LEAP	1x interview
PLE7	LEAP, project staff	2x interviews; PPIE panel
PLE8	Project staff	1x interview; 1x focus group
PLE9	Project staff	1x interview
PLE10	Advisor	1x focus group
PLE11	Advisor	1x interview; 1x focus group
PLE12	Advisor	1x focus group
PLE13	Advisor	1x interview
PLE14	Advisor	1x focus group

### Data collection

Two methods of data collection were used: (i) in-depth, semi-structured interviews and (ii) focus groups (FGs). Both were selected because of their potential to elicit rich storytelling from participants, and in recognition of the role of storytelling within LE activities as an accessible engagement strategy that can facilitate the sharing of testimonies in ways that resonate with listeners by building empathy and challenging preconceived narratives [10, 26]. Interviews have previously been used to understand GRH [44, 45]. The combination of the interviews and FGs was complementary and enabled in-depth investigation of LE involvement in and beyond CAGH, while also capturing the views of people who did not have LE but did have experience of working with PLE in gambling harms reduction efforts. PLE, PS and SS were given a participant information sheet and confirmed their consent to being audio-recorded and quoted. All recordings were transcribed verbatim.

The semi-structured interviews (n-33) were conducted online in two sets: a first set of interviews at the midpoint of the CAGH intervention, and a second set of follow-up interviews at the end-point. Interviews ranged from 40 min to 1 h 27 min, and involved PLE, PS and SS. Three PLE who were interviewed also sat on the PPIE panel. Two PLE and two PS were interviewed twice: at the outset of the evaluation, and later as a follow-up. Not everyone was interviewed (some PLE participated in the FGs only). In total, 13 interviews were conducted with PLE; 16 with PS; and four with SS.

Following the first set of interviews, three FGs were held online to more fully explore the different types of LE activity that had been identified and to discuss how best to facilitate effective LE involvement. FGs had an average duration of 70 min. FG participants were all PLE and included people external to the CAGH intervention who were recruited via purposive sampling, based on their expertise in a LE activity that formed the topic of each FG. The FG topics were:

FG1: LE in campaigning and awareness-raising.

FG2: LE in education and consultancy.

FG3: LE in peer support interventions.

Participants with roles that spanned the above topics were welcome to join more than one FG, resulting in some overlap. FG participants were invited to accept a voucher to thank them for their time and contributions.

### Data analysis

The Framework Method [46] was used to organise data into a framework that informed the onward development of themes [47]. The Framework Method proceeded through an initial categorisation phase to an analytical framework that was then refined. Theoretical perspectives and concepts from the LE literature also informed the analysis [46]. Abductive reasoning, which allows themes to be developed from initial codes for ongoing exploration alongside prior theory, underpinned engagement with the literature [48, 49].

Data were analysed thematically using a coding apparatus collaboratively developed via team coding sessions using NVivo 12, Microsoft OneNote (employing the tagging function to mark-up data extracts), and in-person discussion and diagramming. A first round of analysis was carried out after the first set of interviews and the FGs. A second round of analysis was carried out after the follow-up interviews [50]. Both rounds of analysis fed into the identification of themes in the data.

## Results

Four themes were identified:

Theme 1: Personal journeys to Lived Experience involvement.

Theme 2: The value added by Lived Experience to interventions for addressing gambling-related harms.

Theme 3: Emotional impacts on people with Lived Experience.

Theme 4: Collective Lived Experience and diverse lived experiences.

The themes interlink with each other: for example, Theme 1 shapes the contributions made by LE identified under Theme 2. Theme 3 affects how people navigate the individual journeys of Theme 1, and considers the range of emotions that LE involvement brings to the fore for them and others. Theme 4 encompasses the diversity of people’s journeys and the activities that PLE are engaged in, as well as the importance of diversity in who is represented in LE involvement and the LE community in the gambling harms reduction sector.

### Personal journeys to lived experience involvement: ‘lived experience still seems a little bit of a title’

This theme focuses on the role of personal journeys in how PLE become involved in GRH reduction efforts; the tensions arising from the professionalisation of LE involvement; and the new understandings around addressing GRH that result from LE involvement across informal and formal domains.

PLE become involved in the gambling harms reduction sector through unique, individual journeys that are ongoing and frequently non-linear. Journeys to LE involvement intertwine with people’s ongoing journeys following their experiences of GRH as they develop working relationships in the gambling harms reduction sector, or tap into the spontaneously-emerging networks that underpin the LE community. Without a structured pathway to follow, PLE innovate and craft their own trajectories through this challenging “space”:

*The Lived Experience space is a walking contradiction because it is one of the most inclusive environments to be in, but it is also one of the most difficult to navigate.* (PLE1, FG2)

PLE forge their own techniques and strategies for harnessing their LE to address GRH in meaningful and impactful ways, often in response to gaps in services. For example, one participant described how their LE motivated them to train as a counsellor specialising in GRH:

*the counselling that I experienced was quite outdated and was with people that really didn’t have any idea or understanding of what gambling addiction is […] So, I kind of had to go on my own journey […] And that led me to thinking, ‘Right, [what] would actually be [helpful for] other people’ […] And then [I] set myself up as a private practitioner, initially – or primarily – to work with people who were experiencing gambling harm.* (PLE9, interview)

Informal domains both provide a springboard to LE involvement, and benefit from the agility and freedom of LE to work around bureaucratic “red tape”:

*I’m not saying that people are unprofessional in the Lived Experience space but the things that they can do and add […] the way they’re able to form, means that they can do things that we [formal services] can’t do.* (PLE1, interview)

For some PLE, their personal journeys were intertwined with self-directed efforts to craft roles that reflected past professional experiences and skills; other PLE sought and acquired new professional skills that they could use in their efforts to address GRH. The diversity of past professional experience was viewed positively by the LEAP, and meant that different areas could be covered by different people:

*[We have] experts in certain areas, and there’s areas where I don’t particularly get involved. Like my background was accountancy before, I was an accountant for 12 years, and then there’s someone who works in the NHS, there’s a former copper […] people have their areas of expertise.* (PLE3, interview)

One PLE spoke about their surprise in being able to contribute their design skills in this context. Moreover, while they no longer enjoyed football (because of its excess of gambling adverts), their in-depth knowledge of the sport meant they could respond to a request for information. They reported that it was ‘great’ to draw on past knowledge in this way:

*I created the logo for [VCFSE organisation], so it was good to just use some completely different skills that I didn’t think would come in handy […] I’m [also] on a call next week, someone with [redacted] University’s looking at gambling advertising and football: she just wants to do some sort of research on that. And that was one of my main things, was football and things, so it’s great to have a bit of input on that.* (PLE3, interview)

As PLE in the gambling harms sector become more established, opportunities for formalising their activities into salaried roles are presented. Professionalisation, in both the statutory and VCFSE sector, has positive and negative implications for PLE. Formal posts with training and qualifications attached, such as counselling and peer support roles in NHS gambling services, can be beneficial to PLE in providing recognition in the form of pay, organisational structures that safeguard self-care and personal boundaries, or prevent the burden of work being overwhelmingly devolved on a single individual. The below account describes a personal journey into the LE space that blurred the boundaries between personal and professional social media use, with largely positive results:

*At the beginning I was keen to keep my personal Twitter and my work’s Twitter separate, but […] they started to blend […] That helped my recovery, we all know Twitter and social media can be a toxic space as well, there’s good and bad in Twitter, but my experiences were good in that I’ve built up my network […] it’s got me in employment.* (PLE1, FG2)

But the ‘incremental professionalisation’ (PLE10, FG3) whereby LE becomes ‘a little bit of a title’ (PLE14, FG3), can impose unwanted constraints. PLE needed to know how to fit one’s own (rarely straightforward) LE journey into uncompromisingly linear ways of working:

*you’re on your own, trying to hold all that up and be a representative for peer support […] Lived Experience is not enough in roles like [peer support] because there’s everything else that comes with it, of understanding a data system and learning how to use it and going on six months’ worth of training that you have to [have] to be at a certain level […] so that can be a barrier.* (PLE8, FG3)

Some PLE resisted professionalisation and the titles and training that this entailed, but still sought to be involved. Furthermore, some were concerned that they were being incorporated into statutory health services in ways that was ‘de-professionalising’, suggesting a form of exploitation:

*There’s an economic rationale for including and involving people with Lived Experience because […] it is de-professionalising […] perhaps for economic reasons […] [S]ome of the workload and responsibilities have been pushed onto volunteers and lower roles […] I don’t want to use the word ‘exploitation’: I will use it though. I’ll use the word ‘exploitation’ softly. There was a sort of creeping ‘taking advantage of’: exploitation.* (PLE10, FG1)

### The value added by lived experience to interventions for addressing gambling-related harms: ‘I see gambling adverts for what they are’

The value of LE is linked to its variety: it is ‘not just one thing’ (PLE3, FG1). LE contributions to public health span two complementary categories of activity that PLE undertake: supporting people, and driving social change. This theme provides examples of the value LE adds to both sets of activity in informal and formal domains:

1. Supporting people – Informal support occurs through spontaneously emerging friendship networks and relationships, with people “being there” for each other when in need. Formal support can include peer support and counselling roles through which PLE help and mentor others, including roles for the NHS and other treatment services.
2. Driving social change – Social change efforts can occur through informal campaigns by PLE to hold people and organisations to account for GRH and to raise awareness. Formal roles include advocacy, consultancy and education, including involvement in forums to advise public agencies, research panels, and educational and training initiatives.

Activities overlap across each category. For example, campaigning can include a form of peer support that involves ‘Lived Experience helping Lived Experience’ (PLE1, interview). In treatment and support interventions, LE’s value resides in the shared experience of harms, and the empathy and understanding that such sharing entails can help efforts to effect social change.

LE involvement in therapeutic interventions can negate the personal impacts of the shame and stigma surrounding GRH, making it more likely that people experiencing harms will engage with peer support, mentoring or counselling. LE involvement may remove the fear of judgement, facilitating people to open up about GRH. PLE provide positive examples of recovery in therapeutic interventions:

*If somebody’s got Lived Experience of that, okay, I’m going to be more inclined to access that […] I’m going to be more inclined to buy in to the process.* (PLE9, interview)

The value of LE in educational and training interventions lies in the understanding of GRH that LE contributes, and the means of engagement through which awareness is raised. By sharing their stories, PLE can humanise GRH statistics, or make the harms experienced by individuals relatable for people who have not had those unique experiences. Storytelling can be tailored to suit the purpose of the encounter and the audience: a careful balance between seeking maximum impact, and avoiding additional harms (stories featuring suicide, for example, may need to be adapted before they can be shared). One LEAP member described the value of their input in an educational intervention that showed their LE story on film while they were physically present in the room:

*And that film is loosely based on my story and I’m in the film […] And it is a nice moment when the kids in the room watch the film […] and, all of a sudden, my face pops up and I start saying, ‘Oh, yes. I had a really good upbringing’ and then they all look and realise, ‘Oh, it’s you. That’s why you’re here. I get it now.’ […] When I’m actually delivering it, I can use my experiences to expand on the stuff that’s seen in the materials and in the film. And I can answer questions as well. Sometimes, they do ask ‘How much did you lose?’ or ‘What should I do if I’ve got a problem?’ And people without Lived Experience can answer the last question, maybe, but, perhaps that question is always answered better by someone who knows, who has lived it. And that is the value of Lived Experience to me: is that insight: that unique insight.* (PLE7, interview)

Storytelling combines effectively with developing GRH “literacy”, or critical health literacy: a skillset that equips people to critique if and how messaging serves industry interests, and take action to address the wider determinants of health associated with gambling. Strong counter-industry narratives featured in LE accounts. This, a public health professional observed, was unique to the sector and aligned with a public health approach:

*the gambling lived experience community actually, in my opinion, are advocating for quite upstream measures around gambling, which isn’t always what people are looking for, say, from the drug and alcohol perspective. My experience is that people are more advocating for treatment and better access to treatment. Which, in gambling, there’s a need there as well but what people are actually asking for is much more upstream. Which is quite interesting and slightly different from other areas. And, obviously, those upstream interventions align very closely with what you’d want from a public health perspective.* (SS1, interview)

The following data extracts show how such counter-industry framings can enhance critical health literacy, here enabling people to withstand the pull of gambling advertisements:

*I didn’t even really know that these products were designed to guarantee profit, I just thought I could win […] that sort of [counter-industry] messaging if I’d have seen that, would have had more impact than just hearing my own story […] If I’d have heard my Lived Experience story at 16, 17, I’d have probably said, ‘Oh well, unlucky but I know what I’m doing still’.* (PLE7, interview)

Development of critical skills in relation to gambling was built-in to some educational interventions, for example one in which a slot machine is dismantled to demonstrate its inner (rigged) workings. Involvement in CAGH also contributed to developing a critical mindset in the case of a community project that had been promoting a charitable lottery in its email signature, but subsequently recognised this as being ‘contradictory with the aims of the gambling [harms reduction] project’ (PS4, interview).

A shared LE understanding of the importance of upstream interventions was, however, not echoed in a shared perspective on strategy – notably on whether PLE should work with the gambling industry. This issue remained contentious and emotive for the LE community, with different perspectives apparent:

*[gambling] is a business, it is there to make a profit, but it’s how they make that profit and who they make that profit from, that’s where the argument sits for me. And so that’s why I think dialogue and conversation with the industry is important because those that are in the Lived Experience community that complain about people talking to the industry, working with the industry, well, I’ve no time for that because what’s the point, if you’re not going to have a conversation with the industry you’re always going to be poles apart and there’s never going to be any progress, you have to have conversation.* (PLE1, interview)

*I wanted this project as a whole to really disrupt, and one of the disrupting things would be that we won’t work with any organisation directly funded by the gambling industry and that’s not happened with this.* (PLE7, interview)

### Emotional impacts on people with lived experience: ‘it was like I’d given blood and never had anything to replenish me’

LE involvement in gambling harms reduction efforts exerts ongoing emotional impacts on PLE. Conflicting emotions are raised, which can range from frustration with policy inertia and having their experiences contested following media appearances, to more positive impacts: a sense of purpose and fulfilment, hope and passion for change, empathy and emotional intelligence, and new understandings of how to address GRH. This theme describes the emotional impacts on individual PLE who contribute to this sector, and the personal and professional benefits that can accrue.

PLE expend considerable personal time and effort in LE involvement activities around GRH, which can make such involvement exhausting. Exhaustion is exacerbated when organisations engage with PLE superficially, or when responsibilities are devolved on individuals without adequate support in place:

*that’s why I left my [peer support] role […] it was like I’d given blood for nine or ten months and never had anything to replenish me.* (PLE1, FG3)

To safeguard PLE’s personal resources of self-care from depletion, supervision – as practised in counselling – and peer-to-peer support is important in providing space for individual reflection and replenishment. In the absence of this, the ‘goodwill’ on which LE involvement is run can assume an ever-present availability on the part of PLE. This erodes the professional boundaries put in place to protect PLE’s energy and time, because ‘you’re not going to say ‘Sorry, can you ring me back during office hours’: that isn’t how it works’ (PLE1, FG2):

*there is a risk sometimes that you forget that you yourself are in recovery because you spend so much time focused on other people and trying to help them – and quite rightly, because you do that because you want to do [it] – but you can sometimes get this feeling of invincibility. Not a conscious one, but subconscious. You can almost forget that, hang on a minute, this still applies to me.* (PLE1, FG2)

*you’ve been that person with no one to pick up the phone to […] That’s what the Lived Experience gives you, because it is those things where you think, I know they need me on the phone right now […] There are people we know who are living and breathing today because they’ve had those 3am calls, because people like us have not respected our boundaries.* (PLE1, FG3)

The authenticity of LE, of value in disseminating LE insights, is also what makes PLE potentially vulnerable: ‘it brings that emotion, your voice will crackle and stuff like that, that is what it is: it’s authentic’ (PLE11, FG2). While LE involvement can provide a sense of meaning and purpose to PLE, it can also be emotionally and physically draining if adequate support is not in place. Adequate support and safeguarding are, therefore, vital to protect PLE and sustain their involvement:

*If people are recounting their story and bringing up, in some cases, quite painful memories, then they really should then have somewhere that they can go, where they can check themselves out and make sure they’re safe […] I almost feel a little bit worried for people if they are recounting their story on a daily basis and bringing up things that maybe are still unprocessed or uncomfortable and they haven’t got anywhere to take that.* (PLE9, interview)

Some PLE in the sample were active campaigners who advocated for the adoption of a national public health framework. They reported that such campaigning could be extremely challenging, with their stories being frequently contested by people hostile to arguments for industry regulation – adding to the emotional pressures on PLE. Some believed that this was unique to the gambling harms reduction sector because, they argued, LE perspectives are not scrutinised or challenged in the same way in other sectors, where there is less campaigning among respective LE communities for upstream national policy reform. One PLE stepped back from campaigning because of this pressure, but sought to support those who continue to campaign and urged them to engage in self-care:

*[I]t’s okay to take a step back […] I admire the [names of prominent gambling harms campaigners] who are relentlessly out there. You can be attacked if you’re out there, you can be criticised, you can be judged […] If someone does say […] [to me], ‘At the moment this is really tough: I’m feeling this is impacting me personally’, the first advice I give to that person is ‘Think about you and only you’ […] It’s not being selfish […] [to be] mindful of your self-care.* [PLE11, interview]

Differences in perspective on whether to engage directly with the gambling industry created internal tensions within the LE community, sometimes resulting in intense disagreement on social media. At the root of this issue was a debate about the funding of services for addressing GRH, with some PLE contributing to gambling industry-funded treatment services and educational campaigns:

*[T]he biggest one is the funding element of it all […] The reason that it can create some conflict is because a lot of [people with] Lived Experience are out there working […] [for] organisations [that] are funded through the industry. I have no views on it at all in the sense of [how I view] those individuals and what they do, but that can create conflict within Lived Experience […] [P]eople can take offence and then it creates these divides. The only people that are loving that is the industry itself.* [PLE11, interview]

### Collective lived experience and diverse lived experiences: ‘a collective voice of lived experience is better than a singular one’

As a term, ‘LE’ is frequently discussed as if it constitutes a single, authoritative experience. This is at odds with the plurality of perspectives that together create the LE community: diverse *lived experiences* which, in their variety, provide the whole with a greater potential influence and legitimacy than could be achieved by individuals alone. This theme considers the role of LE in representing, and doing justice to, the diversity of people’s experiences (including diversity of ethnicity, gender, extent of harm, and funding streams).

The lack of ethnic diversity in LE within the gambling harms reduction sector is significant, and visibility of some populations over others can undercut the relatability that is central to the power of LE: ‘all the Lived Experience panels that I’ve been involved in are just 90% white men […] it does restrict you in getting to those other communities and hearing other voices’ (PLE7, interview). There is recognition in the data of the risks involved in over-reliance on individuals, and on single accounts of LE, at the expense of scalable interventions:

*the results that we get are better when it’s someone with Lived Experience [delivering] […] but it is quite a big worrying question that I think about a lot, is have we created a programme that focuses so much on me but what do we do when I’m not doing it?* (PLE7, interview)

In this sector specifically, it is important to recognise that, beyond diversity in the communities affected by GRH, diversity also has broader relevance. Diversity in this context therefore includes the diverse forms of gambling that are available, ‘in a betting shop or a casino or online slots’ (PLE1, interview); the diverse harms associated with gambling; and the diverse strategies mobilised to address those harms:

*There is another point of diversity that I think is more important than actual diversity [of protected characteristics]: is the diversity of experiences […] gambling is changing. And I don’t think we’re reflecting new people’s experiences […] like the rise of day trading, the rise of crypto, the different products, the different practices of the industry, the way the industry’s regulated. I think we need to say up-to-date with what’s going on, and that involves bringing in the people with Lived Experience who are more current. So diversity’s not just about gender and religion and race – whatever – it’s about experiences too.* (PLE7, interview)

There is a role for public health in cultivating connections with diverse communities to facilitate their LE involvement. Diversity in GRH means more than a *collection* of representative “voices” (‘we are more than our stories’: PLE7, interview) – it also supports a community or *collective*, within which there is room for diverse experiences of GRH and diverse viewpoints, including critique of LE involvement itself. As one participant expressed it, ‘a collective voice of Lived Experience is better than a singular one’ (PLE11, FG2). This distinguishes LE in the collective sense (‘collective LE’), from LE understood as encompassing a range of perspectives. Tensions can arise between the two, particularly around the divisive question of whether to engage with industry:

*you need to have those conversations with [the gambling] industry to say, ‘this is the reality’ […] There needs to be more cohesion and more conversations and understanding that each other exists, and each other has a value and a reason to exist and not just an outright ‘No, we’re not going to engage’.* (PLE14, FG3)

Some PLE may be open to ‘a healthy two-way discussion’ (PLE13, interview) on such tensions, but the structural power imbalance in decision-making in this space undermines diversity of opinion:

*you can give a perspective that nobody else can give [but] I think sometimes it’s just been used as a bit of window dressing – oh, we’ve got a Lived Experience group, but they’re not given any authority to make decisions […] I was part of a four-person Lived Experience panel that was scoring bids for tender […] and our four votes only counted as one vote, so they watered down what we did.* (PLE13, interview)

Of note here is that ‘window dressing’, used here to refer to tokenistically involving PLE, is distinct from another image used in the data: the ‘tick box exercise’ (LE3) whereby LE involvement is viewed as something to wheel in and “get done”, before swiftly moving on: ‘just been brought in to tick a box or to be consulted on, and then nothing goes anywhere’ (SS1, interview).

## Discussion

### Contributions of lived experience to a public health approach for reducing gambling-related harms

The key insight from this evaluation is that involving LE can contribute substantially to a PH approach to gambling harms reduction, and in varied ways. The themes outlined above demonstrate how LE contributions contend with tensions in the application of LE involvement. LE is really many lived experiences, as different domains and activities related to LE involvement give rise to different contributions and challenges.

**Fig. 1:**
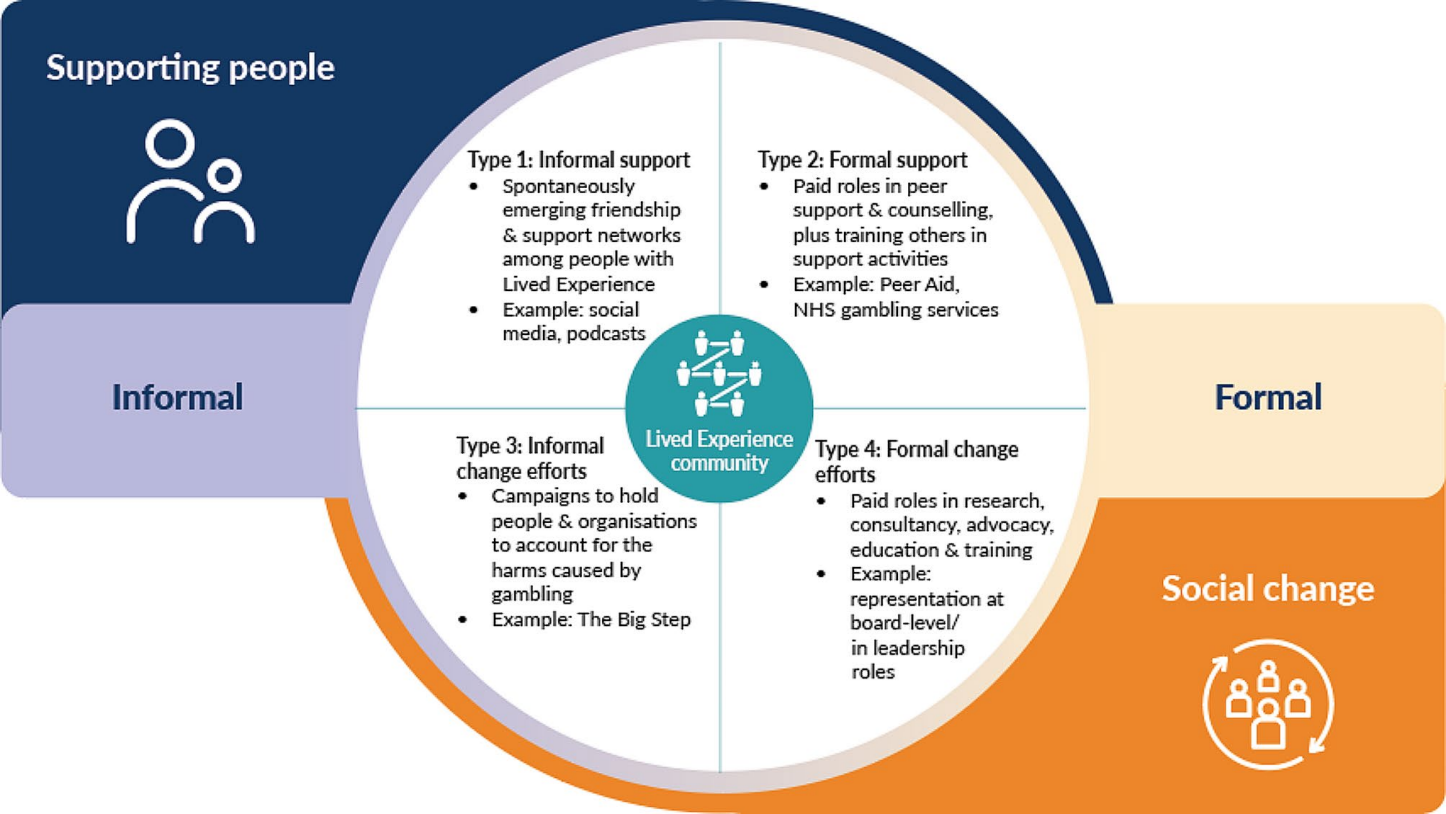
A typology of lived experience activities in the gambling harms reduction sector

The themes confirm and add to the existing literature on LE in public health approaches to GRH reduction. They demonstrate that a range of participation options for PLE in the GRH sector is important [51], and they develop understandings of the positive and timely contributions that LE involvement can make to guide public health interventions. To aid the identification and harnessing of these contributions and their implications for public health, a typology of LE involvement specific to the aims associated with gambling harms reduction based on the data has been developed and is presented below (Fig. 1).

PLE undertake many activities that support public health. Some of these are delivered through formal job roles that offer opportunities for PLE to utilise their unique insight and motivation to support people or affect social change. Underpinning formal involvement, there are also self-organising networks in which people support each other and engage in social campaigns to deliver change. Figure 1 maps these activities. Broadly speaking, the activities have two core purposes or ‘functions’: (a) supporting people and (b) social change. The form that these activities take on, however, changes depending on whether they are enacted in informal or formal domains. Thus, the figure presents a four-fold typology of LE activities relevant to public health. The four types are fluid and can overlap with each other. Examples of each of the types in practice are provided.

The typology visualises the interrelated activities that PLE undertake across informal and formal domains in support of a public health approach to reducing GRH. These activities contribute to supporting people and driving social change, and are steered by the agency of PLE. Each forms part of the contributions that PLE make to GRH reduction efforts. The various activity types have an impact, both on the person “doing” LE and those who “receive” it. Immersion in spontaneously emerging friendship and support networks can help people through the sharing of experiences, understanding, empathy and hope. Similarly, for PLE who are involved extensively in the field, formal job roles provide opportunity for recognition, pay, meaning and purpose. Each activity type can present challenges, whether it is located in informal or formal domains.

**Fig. 2:**
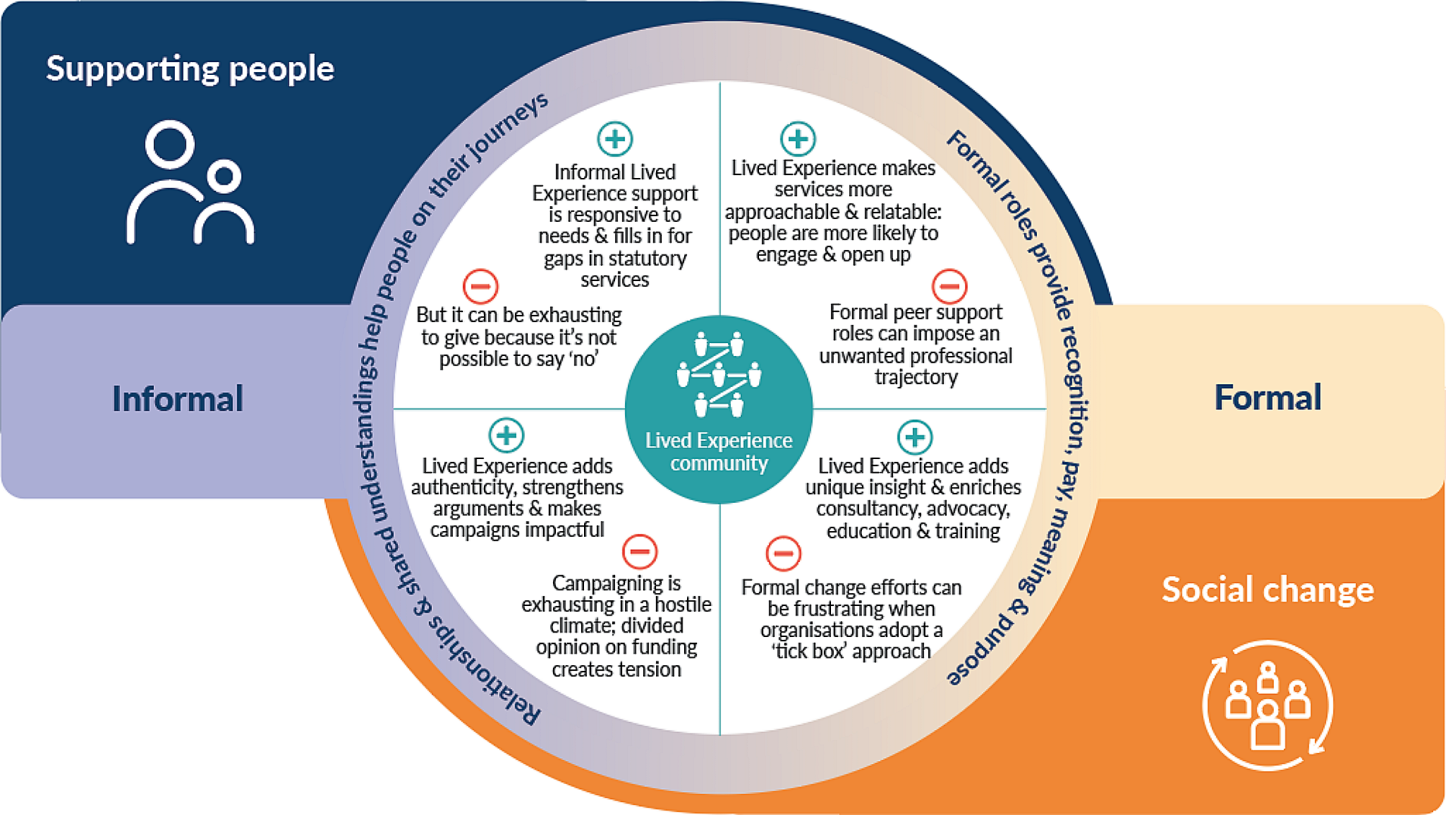
A mapping of the positive and negative impacts of LE involvement in the gambling harms reduction sector

Figure 2 follows sequentially on from Fig. 1 in mapping the impacts (positive and negative) of LE involvement, and the emotions such involvement raises in PLE. It also considers the different skills developed (in critical health literacy, media training, managing commercial and political tensions) and deployed across informal and formal domains. As in Fig. 1, the importance of diversity in the LE community is central.

Figure 2 presents the positive and negative impacts of LE involvement. LE adds something distinct to the activity types in Fig. 1: it makes formal services approachable, and it adds authenticity to social campaigns. But there are corresponding possible negatives for the person. These include exhaustion from filling in gaps in statutory support services, as it is not easy to decline requests of help from people in need. Social campaigns take time and are frustrating when policymakers and senior leaders do not respond to evidence-based demands (e.g., to end gambling sponsorship in sport), or treat LE voices as ‘window dressing’, rather than genuinely listening to their views. As shown in Sect. 3.3 and 3.4 above, there are also differences of opinion among the LE community on strategic questions: some PLE urge engagement with the gambling industry and work for, or receive funding from, gambling operators, while others emphasise the importance of independence from industry and aspire to work for or with organisations that are funded from independent sources. This can lead to tensions within the LE community.

Involvement in LE requires support to assist PLE to manage the associated tensions as well as continuing personal and professional development (CPPD) opportunities that accompany the translation of local experiences for use in wider contexts. Safeguarding is necessary to sustain the life-blood of LE involvement and to harness and repurpose the emotions associated with it for positive ends while continuing to protect PLE who contribute their experiences. The development of formal pathways to LE involvement is challenged by the encroaching professionalisation of LE work and the narrowing of the expertise of PLE to a single subject. The “vitality” of LE that is threaded throughout the data – the energy and responsiveness in how LE is spoken about, and how its value is perceived – is sapped by institutional workflows that require PLE to somehow fit the messiness of LE into linear career trajectories. At the other end of the scale, informality can lead to eroded boundaries and burnout for emotionally-exhausted PLE.

Both figures prompt consideration of what public health can do to enhance the positives and lessen the negatives, while retaining the value of LE’s informality. The perspectives of PLE can contribute important knowledge to inform a social harms perspective on gambling and insights into how interventions to address GRH can be designed and delivered effectively [17]. A LE collective that welcomes and accommodates people’s diverse lived experiences and expertise beyond their LE is needed. Such a collective might best be facilitated by a public health approach to reducing GRH that supports PLE and public health professionals to develop their and others’ critical health literacy relevant to gambling. The entanglement of LE involvement in gambling harms reduction efforts with the commercial determinants of those harms complicates LE in this sector by implicating it in continued health-harming industry operations [28].

LE encompasses, and should do justice to, diverse experiential knowledges. LE spans unique, personal, and ongoing journeys that can be shared to advance gambling harms reduction, chiefly through two complementary areas of work: supporting people and driving social change. For LE involvement to be effective, full consideration must be given to the emotional impacts of LE roles and the multiplicity of experiences being lived through by those who take on such roles. LE contributes a valuable alternative way of looking at things that public health needs to learn from and use if it is to accommodate non-linear LE journeys and facilitate the translation of LE for public health purposes. A collective LE stance is needed that can overcome differences of opinion on strategy within the LE community, while also recognising the value of this plurality and the reflections it prompts on the part of PLE and the organisations that they advise.

### Implications for lived experience involvement in public health

This study has implications for what LE involvement in public health should look like to ensure that it is sustainable, fully-supported and applied to its full potential. It is vital that public health professionals value the existing expertise of PLE and LE-led organisations specialising in GRH to help facilitate work in this area. Done right, the involvement of PLE is meaningful and can make a difference.

In the UK, the proposed adoption of a statutory levy to fund work in the gambling harms reduction sector provides a recent example of why listening to PLE is important: some public health actors critique the levy because of its continued links with industry [50], but more work is needed to understand whether this reflects the differing viewpoints of PLE.

The evaluation team consulted the PPIE panel, which included PLE of GRH, about a set of recommendations from this evaluation. Many of the recommendations align with previous research [26], but the following are additions specific to this research:

- Organisations new to working in this space should proactively reach out to LE-led organisations to learn from them how to facilitate LE involvement, with co-design built-in from the start.
- Professionalisation of PLE should be aligned with individuals’ goals and professional and personal experiences outside of gambling-related LE. Organisations have a responsibility to nurture the potential of PLE in ways tailored to individuals’ skills and motivations, rather than imposing a standard training pathway.
- Support for PLE should be designed to help people manage the emotional toll of LE involvement. This could look like dedicated, protected time for regular supervisions with PLE to check-in, discuss wellbeing, and set goals.
- LE involvement should be more than a ‘tick box exercise’, or ‘window dressing’: active LE involvement and integration in diverse intervention types and authority in decision-making processes should be the standard.
- Recruitment and routes into LE involvement should be diverse to ensure that communities affected by GRH are equitably represented. Public health actors need to help the LE community to become more diverse by connecting with diverse communities.
- National government should establish independent, transparent and sustainable funding arrangements for gambling harms prevention and treatment that embed LE oversight.
- National funding arrangements should ensure that LE-led VCFSE organisations have access to sustainable funding in recognition of their contributions and expertise, including in education and treatment, developed in the absence of statutory public health and NHS funding.

### Limitations

Our sample of PLE was not diverse. This reflected the reality of LE involvement on the ground, as the PLE who participated in the gambling harms reduction intervention were predominantly White British. As we explore in theme 3.4, this local weakness of representation was recognised, and the PLE sought to connect with and recruit from diverse ethnic groups from the region. The public health professionals involved in CAGH had a role in this, as they arranged opportunities for the PLE to speak at community and faith events, but ultimately it proved challenging to recruit. Ensuring diversity in LE involvement should, therefore, be viewed as an ongoing objective and future research should prioritise efforts to recruit a diverse sample to explore the barriers to LE involvement.

PLE who were motivated to participate in this study also did not represent the spectrum of harms engendered by gambling, and tended to be individuals with previous experience of LE involvement (education, training, peer support), rather than those less-experienced or new to this sector [26]. Stigma significantly affected recruitment, particularly of community project participants, given the LE community around GRH in the UK is of a size that individuals are potentially identifiable [32].

The decision not to hire PLE to conduct the interviews or coordinate the FGs is a limitation: a peer-interviewer approach, with trained PLE interviewing PLE, could have helped reassure participants as well as providing a way to give back to the LE community [52]. The PPIE panel were however consulted throughout the project, and a free training session in evaluation methods was offered to interested participants post-intervention.

Research based on LE can face difficulties in claiming to be generalisable, because LE is so unique [29]. However, foregrounding LE, as this evaluation aimed to do, can offer lessons for other public health systems by drawing comparisons with industry-led versions of events [29].

## Conclusion

This study has highlighted the importance of integrating LE into public health approaches to address GRH. Public health professionals should ensure that LE is included in intervention design, delivery and evaluation processes, and that support is in place to facilitate LE activities related to reducing GRH. Harnessing the contributions of LE in all their diversity is essential to inform the direction and responsiveness of the gambling harms reduction sector in the UK as it develops at pace.

A LE-informed public health approach to gambling harms reduction requires local access to involvement for PLE via diverse and equitable routes that are free from stigma and that can contribute to decision-making. LE involvement in this sector needs to enable PLE and people without LE to develop the affective and critical skills necessary to navigate the tensions inherent in co-existing with industry-funded LE involvement programmes.

## Data Availability

No datasets were generated or analysed during the current study.

## Abbreviations

AO: Affected Other
CAGH: Communities Addressing Gambling Harms
CPPD: continuing professional and personal development
FG: focus group
GRH: gambling-related harms
LE: Lived Experience
LEAP: Lived Experience advisory panel
PLE: person with Lived Experience, people with Lived Experience
PPIE: Patient and Public Involvement and Engagement
PS: project staff member
SS: senior stakeholder
VCFSE: Voluntary, Community, Faith and Social Enterprise
